# Development of a Tremor Detection Algorithm for Use in an Academic Movement Disorders Center

**DOI:** 10.3390/s24154960

**Published:** 2024-07-31

**Authors:** Mark Saad, Sofia Hefner, Suzann Donovan, Doug Bernhard, Richa Tripathi, Stewart A. Factor, Jeanne M. Powell, Hyeokhyen Kwon, Reza Sameni, Christine D. Esper, J. Lucas McKay

**Affiliations:** 1 Jean and Paul Amos Parkinson’s Disease and Movement Disorders Program, Department of Neurology, School of Medicine, Emory University, Atlanta, GA 30322, USA; msaad3@emory.edu (M.S.);; 2 Department of Neuroscience, Georgia Institute of Technology, Atlanta, GA 30322, USA; 3 Department of Neuroscience and Behavioral Biology, College of Arts and Sciences, Emory University, Atlanta, GA 30322, USA; 4 Department of Psychology, Laney Graduate School, Emory University, Atlanta, GA 30322, USA; 5 Department of Biomedical Informatics, School of Medicine, Emory University, Atlanta, GA 30322, USArsameni@dbmi.emory.edu (R.S.); 6 Department of Biomedical Engineering, Georgia Institute of Technology, Atlanta, GA 30322, USA

**Keywords:** motion capture, Parkinson’s disease, essential tremor, machine learning, support vector machines, XGBoost

## Abstract

Tremor, defined as an “involuntary, rhythmic, oscillatory movement of a body part”, is a key feature of many neurological conditions including Parkinson’s disease and essential tremor. Clinical assessment continues to be performed by visual observation with quantification on clinical scales. Methodologies for objectively quantifying tremor are promising but remain non-standardized across centers. Our center performs full-body behavioral testing with 3D motion capture for clinical and research purposes in patients with Parkinson’s disease, essential tremor, and other conditions. The objective of this study was to assess the ability of several candidate processing pipelines to identify the presence or absence of tremor in kinematic data from patients with confirmed movement disorders and compare them to expert ratings from movement disorders specialists. We curated a database of 2272 separate kinematic data recordings from our center, each of which was contemporaneously annotated as tremor present or absent by a movement physician. We compared the ability of six separate processing pipelines to recreate clinician ratings based on F1 score, in addition to accuracy, precision, and recall. The performance across algorithms was generally comparable. The average F1 score was 0.84±0.02 (mean ± SD; range 0.81–0.87). The second highest performing algorithm (cross-validated F1=0.87) was a hybrid that used engineered features adapted from an algorithm in longstanding clinical use with a modern Support Vector Machine classifier. Taken together, our results suggest the potential to update legacy clinical decision support systems to incorporate modern machine learning classifiers to create better-performing tools.

## 1. Introduction

Tremor is defined as an “involuntary, rhythmic, oscillatory movement of a body part” and is the most common human movement disorder [1]. It is a feature of many neurological conditions [2] and can also result from various causes such as trauma or side effects of medications [3]. For example, a characteristic sign of Parkinson’s disease (PD), the second most common neurodegenerative disorder worldwide [4], is a tremor that appears while at rest (often a “pill-rolling” tremor of the thumb and forefinger) [2]. Essential tremor, however, is a primary disorder of tremor and is roughly eight times more common than PD [5]. Furthermore, some other oscillatory movements exist that are not tremor. Myoclonus, for example, results in rapid, brief movements, and dystonia produces sustained or intermittent muscle contraction causing abnormal movement, postures, or both; either of these conditions can present with movements resembling “tremor” [2].

Currently, tremor disorders are diagnosed clinically based on skilled observation by experts; progression is gauged with standardized clinical scales based on carefully instructed movements designed to bring tremor into evidence. Quantitative measurements are approximated by eye, and there is no automated clinical decision support. Clinicians characterize the features of the tremor, including body distribution, position when it occurs, provocative factors, frequency, gross amplitude, and other possible association neurological signs, and aggregate this information with other medical testing results to identify underlying causes and to evaluate potential treatment plans [4]. In PD and Essential Tremor (ET), overall tremor severity is measured using standardized clinical scales like the Movement Disorder Society-Unified Parkinson’s Disease Rating Scale Part III (MDS-UPDRS-III) [6], the Fahn–Tolosa–Marin Tremor Rating Scale (FTM) [7], and The Essential Tremor Rating Scale (TETRAS) [8]. These clinical scales give general guidelines for tremor amplitude assessment by eye but are not intended to be used with actual measurement tools (e.g., with calipers or an anthropometer).

Recent progress in human activity recognition [9] and edge computing [10] suggest that there is significant potential for automated clinical decision support tools in tremor measurement. Despite this potential, technologies for identifying tremor have progressed slowly towards standardization and clinical uptake [1]. In the research domain, various technologies measure human motion, including body-worn sensors [2,8,11], 3D motion capture [12,13], and, most recently, pose recognition from monocular video [14,15,16]. Digitizing tablets are often used for assessing tremor during tasks like spiral drawing [17,18] and for discriminating tremor from bradykinesia during finger tapping [19]. In fact, the recognition of the potential for spectral analysis in assessing tremor dates back to the mid-1960s [20], and differences in tremor frequencies across disorders have been acknowledged for over two decades [2]. Substantial domain knowledge (and in some cases, cultural) gaps between clinicians and engineers further hamper widespread adoption. Further hindering adoption are the initial set-up costs for specialized equipment, particularly in smaller centers where research is not a primary focus. This is in contrast to fields like cardiology, where automated clinical decision support systems thrive due to large public datasets enabling annual improvements in anomaly detection [21,22].

In our center, we perform comprehensive behavioral testing using 3D kinematic motion capture to objectively evaluate abnormal movements in patients with PD, ET, and other conditions [23]. Indications for this procedure include diagnosis adjudication, identifying rare tremor types, and evaluation for functional neurosurgery, among others. Our behavioral testing paradigm involves multiple standardized upper limb tasks designed to elicit tremor under provoking conditions of rest, posture, and action. Since 2014, we have performed >1500 behavioral tests using analysis pipelines that were developed organically based on clinician domain knowledge without formal evaluation.

A challenge encountered in evaluating tremor analysis algorithms is imprecision in the “ground truth” criteria for tremor presence outlined in clinical scales [6,7,8]. For instance, the MDS-UPDRS-III criterion that “tremor is present but less than 1 cm in amplitude” (corresponding to a score of 1) is perfectly clear for a human rater but poses substantial ambiguity for a machine. Questions arise in implementation, such as the following: (1) along which biomechanical axis or axes should the amplitude be measured, and (2) what size of tremor meets the threshold for being considered “present”?

The objective of this study was to compare tremor detection algorithms developed by our clinic, focusing on their effectiveness in identifying tremors within 3D kinematic data of patients with movement disorders. Performance was compared to ground truth labels that were recorded in contemporaneous notes by clinicians. These labels are straightforward: the tremor is either present or absent. The main goal was to identify the most accurate algorithm for detecting tremor presence or absence in individual body parts during testing sessions.

## 2. Materials and Methods

### 2.1. Data Sources

We compared algorithm performance using a database of 2272 recordings created during standard clinical exams of a convenience sample of N = 52 clinic patients. Patient records were arbitrarily selected by a data abstractor “at random”, but no formal random sampling was used. Clinician records for each patient comprised separate annotations of tremor presence in each of 16 separate extremities in each of multiple trials. Aspects of the testing paradigm have been described previously [12,23,24]; more detail is provided below. In 43 patients (86%), the primary diagnosis was either PD or ET. Detailed demographic and clinical characteristics were available for 50/52 patients. Demographic and clinical characteristics for these patients are shown in Table 1.

### 2.2. Behavioral Testing Paradigm

**Figure 1:**
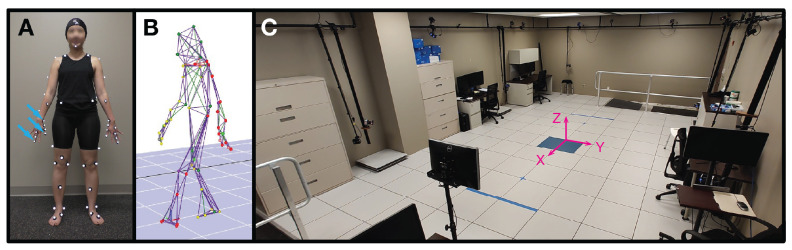
Clinical motion capture facility. Our center uses a custom set of 60 retroreflective kinematic markers for most cases. Markers on the hands (blue arrows on panel (**A**)) enable tremor measurement. From top to bottom, the markers highlighted are R.Wrist, R.Thumb.M3, and R.Finger3.M3 ((**A**); see Table A4 for more description). After data collection, analysis is performed using a de-identified “wire frame” representation of the individual, preserving privacy (**B**). Our 650 square feet center is used for both clinical and research applications (**C**). The origin of the kinematic coordinate system is superimposed.

Behavioral testing was captured through 3D optical motion capture with 60 reflective markers on standardized bony landmarks during a 1-h clinical assessment in our facility (Figure 1). Assessments were billed under Current Procedural Terminology (“CPT” [26]) codes 96000, 96001, and 96004. All patients with Parkinson’s disease were asked to hold their antiparkinsonian medications for at least 12 h prior to the study visit (the practically defined OFF state [27]). At the time of testing, the average time since the last medication dose was 13 ± 5 h. Tasks were designed to provoke various tremors including goal-directed upper limb movements, static postures, and walking [4]. For instance, seated finger-to-nose pointing with the right arm while the left hand is resting on the left thigh (coded sit-point-right or sit-point-1 in data files) aimed to elicit action-provoked tremor in the right upper extremity and rest or postural tremor in the legs, left upper extremity, torso, head, and neck (Table A2). On average, kinematic data recordings were 27 ± 9 s long and ranged from 3 to 92 s, with the shorter recordings generally being overground walking trials in participants with mild symptoms and the longer predominantly being upper limb pointing tasks in more affected participants.

### 2.3. Kinematic Data Recording, Processing, and Export

Data were captured using a 3D motion capture system (Motion Analysis Corporation, Rohnert Park, CA, USA) with 14 cameras recording at 120 Hz. Following testing completion, clinic staff manually postprocess kinematic data using standard interpolation features in Motion Analysis Cortex software (Version 10) for quality control. Occasional low-pass or similar filters were applied on an as-needed basis to address noise in individual markers, but no consistent additional filtering occurred. Each recording’s kinematic data were exported into a standard *.trc tabular format. A typical .trc file for a 30-s recording at 120 Hz comprises 3600 rows (30 s × 120 Hz) and 180 columns (60 markers × 3 axes) of kinematic data. Due to changes in marker labels and occasional missing data, each .trc file was divided into separate .csv files for each body extremity in the accompanying dataset. These files are compatible with standard Python, R, Matlab, or similar software libraries. Summaries of the contents of example files are provided in Table A1.

### 2.4. Annotations

Annotations were taken contemporaneously during the exam for the clinicians’ own use while preparing exam notes. Because tremor is intermittent in nature and typically does not appear across more than a few isolated body regions, annotations typically included separate entries for specific body parts during each recording. For example, the annotation “Left hand: present, F3 and thumb” was used to indicate that tremor was present on the third finger (F3) and thumb of a particular trial. Therefore, the annotations were converted by the study team into separate annotations for each body extremity during each recording. For example, “mild bilateral rest hand tremor” was converted into the annotation “tremor present” for each of the left and right hands. As the presence or absence of tremor in other body extremities was ambiguous in this case, no annotations were provided for other body extremities. In cases where the absence of tremor was described in the original notes (“this gentleman does not have tremor”), tremor was labeled as “tremor absent” for all extremities. In some records (ten trials in two participants), dyskinesias or dystonic posturing were present. These terms refer to abnormal movements that can be misidentified as tremor; these recordings were labeled as “tremor absent”.

### 2.5. Spectral Composition of Kinematic Data

All algorithms used initial preprocessing to isolate spectral (or “frequency-domain”) features of recorded data based on the substantial amount of established research in this area. The majority of parkinsonian and essential tremors typically occur between 4 and 12 Hz [2]—although the spectrum of tremor disorders encompasses a range from 0.5 to 18 Hz [4]. Importantly, tremor is not the only source of frequency-domain energy in kinematic data. In trials that include voluntary movements, like upper extremity reaching tasks, the movements themselves introduce additional frequency components, primarily at lower frequencies (<2–3 Hz). Finally, higher frequency ranges (typically >40–50 Hz) may be prone to artifacts related to aliasing, [28] or other noise, particularly “jitter” in kinematic markers [29]. For this reason, tremor data are typically processed by band-pass filtering. Typical ranges include 1 to 16, 0.5 to 15, or 2 to 30 Hz [1]. All of the tremor detection algorithms examined employed some initial band-pass or other filtering, described below.

### 2.6. Algorithms

Identifying tremor is a process that uses the rich information embedded within motion data from kinematic markers on each extremity to determine whether tremor is present or absent in a particular session. Although this particular set of circumstances is unique, like many general machine learning problems, this process can be broken down into two basic steps. The first step is feature engineering: extracting information (“features”) from raw kinematic marker data. The second step is classifier development: creating a classifier based on the extracted features that determines whether tremor is present or absent. During classifier development, in particular, it is important to perform some hyperparameter optimization to identify the optimal operating point for a given algorithm.

In this study, we compared six algorithms for identifying tremor (Table 2). The first two were developed organically over several years based on clinical expertise and signal processing heuristics. As implemented in our clinic, both algorithms derive engineered spectral features from the kinematic data which are then input into simple rule-based classifiers to determine whether tremor is present or absent. We designated these two as “A1r” and “A2r” as they both used “rule-based” classification methods. While these algorithms were developed iteratively over several years with access to the clinical dataset, no comprehensive hyperparameter tuning was performed, potentially leading to suboptimal parameter settings.

We also implemented two modern machine learning algorithms (B1 and B2) from scratch for this study, both of which (discussed below) combine generic spectral features with modern (as opposed to rule-based) cross-validated classifier architectures. To create a more fair comparison with the modern machine learning algorithms (B1 and B2), we also examined the performance of algorithms A1r and A2r when the features identified by each (summarized in Table 3) were used as inputs to a well-established machine learning model (Support Vector Machines, SVMs [30]) trained and evaluated with 5-fold cross-validation. To distinguish these algorithms from the related algorithms with rule-based classifiers, these implementations are referred to as “A1s” and “A2s”.

The final two algorithms (B1 and B2) were developed specifically for this study based on standard modern machine learning best practices. Both B1 and B2 use basic preprocessing and spectral features together with well-established machine learning models to identify optimal operating points. The details of each algorithm are described below.

#### 2.6.1. Velocity Spectral Peak Detection (Algorithm A1r)

**Figure 2:**
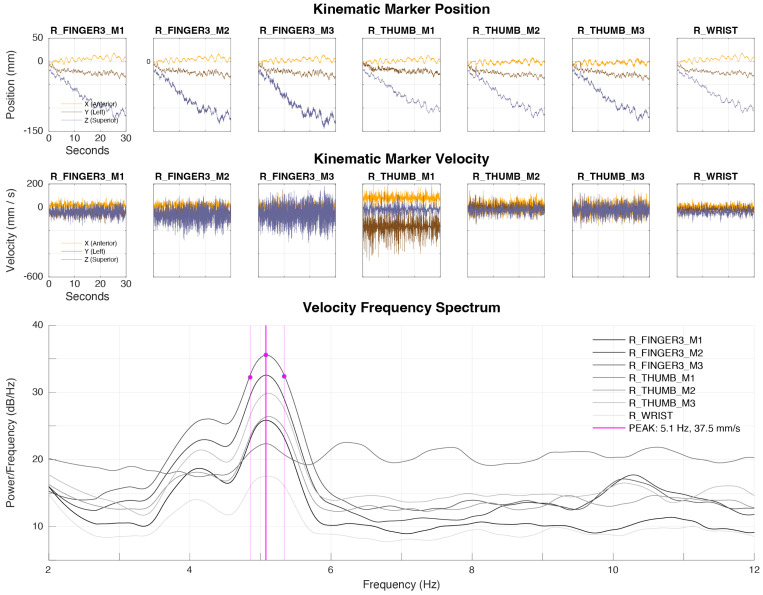
Example of tremor identification with algorithm A1r. Algorithm A1r operates on each kinematic marker on a given extremity and estimates the central frequency (Hz) and spectral power density (db/Hz) of the highest-amplitude tremor observed across markers. Here, the thin lines correspond to individual kinematic markers and pink lines indicate peak values.

The oldest algorithm in use in our center was developed iteratively between 2014 and 2020. The key feature of this algorithm is that it performs numerical differentiation on kinematic data prior to feature identification in the frequency domain. It uses a winner-take-all approach to aggregate tremor features across kinematic markers on a given extremity (described below). An example of tremor identification using Algorithm A1r is presented in Figure 2. This algorithm was implemented in Matlab (Version R2022b; The Mathworks, Natick, MA, USA).

##### Feature Extraction

Raw kinematic displacement data for each marker of a given extremity are zero-phase low-pass-filtered (20 Hz), centered, and passed through a Savitzky–Golay derivative filter to obtain smooth velocity estimates in each of the x, y, and z dimensions. The power spectral densities (PSDs) of the velocity components for each marker are obtained using Welch’s method and combined using the Euclidean norm. The combined PSD of each marker is then converted to log scale, smoothed using a Savitzky–Golay filter, and converted back to a linear scale for spectral analysis. Spectral features are summarized in Table 3. More details on feature calculation are available in the documentation for powerbw.m.

##### Rule-Based Classification

To detect a peak that would indicate the presence of tremor, the peak power and the corresponding center frequency were first detected for each kinematic marker using functionality integrated in the Matlab function powerbw.m. A significant peak should be narrow and symmetric about the center frequency, so any peak with a bandwidth greater than 2 Hz or nonsymmetric power to the left and right of the peak would cast doubt on the presence of a tremor of neurologic origin, which tends to be highly sinusoidal.

Indicators of bandwidth and symmetry are derived using powerbw.m and subjected to threshold rules to determine the presence or absence of tremor. During the development of this algorithm, it was determined that peaks with center frequencies above 10 Hz would also be deemed unreasonable; therefore, central frequencies above 10 Hz are also interpreted as tremor absence. To aggregate features across markers of a given extremity, the algorithm proceeds to detect a tremor on each marker independently. The tremor features for the marker with the largest tremor amplitude on a given recording are used as representative of the entire extremity.

#### 2.6.2. Amplitude Spectral Peak Detection (Algorithm A2r)

**Figure 3:**
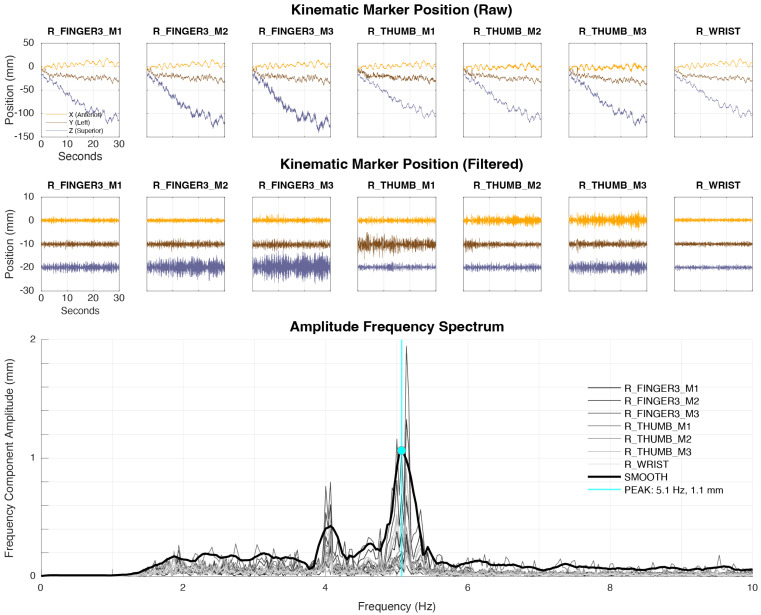
Example of tremor identification with algorithm A2r. Algorithm A2r operates simultaneously on all kinematic markers on a given extremity and estimates the central frequency (Hz) and amplitude (mm) of the highest-amplitude tremor present.

The amplitude spectral peak detection was established in our center primarily to provide tremor identification in the amplitude, rather than velocity domain, in order to enable direct comparison with clinical magnitude cutoffs. The key feature of this algorithm is that it converts all kinematic data from kinematic markers on a given extremity to the frequency domain prior to aggregation with a max procedure. Therefore, the spectral features identified for a given extremity reflect a combination of kinematic markers, rather those of a single dominant marker. An example of tremor identification using Algorithm A2r is presented in Figure 3. This algorithm was implemented in Matlab (Version R2022b; The Mathworks, Natick, MA, USA).

##### Feature Extraction

Raw kinematic data of all markers on a given extremity are high-pass-filtered with a 4th-order Butterworth filter with corner frequency 2 Hz using filtfilt.m in Matlab. The two-sided frequency spectrum is calculated using the fast Fourier transform and converted into the single-sided frequency spectrum of each axis of each kinematic marker. The single-sided frequency spectra of each x, y, z component of all markers on each extremity are combined using a max procedure to create an aggregate spectrum for the extremity that represents the most severe tremor at each frequency. The aggregate spectrum is subsequently smoothed with a Savitsky–Golay 3rd-order polynomial smoothing filter. Frequency peaks in the smoothed spectrum are then identified with the heuristic-based findpeaks.m method in Matlab software using default arguments.

##### Rule-Based Classification

Classification proceeds in two steps. First, the central frequency of the dominant frequency peak identified by findpeaks.m is compared to maximum and minimum threshold values ( <3.5 Hz or >10 Hz, respectively). Peaks with central frequency outside of this range are considered unlikely to be of neurological origin and are discarded. If these conditions are met, the amplitude of the peak is compared to a simple threshold value (0.1 mm) to determine tremor presence. This threshold value was determined over trial and error.

#### 2.6.3. Support Vector Machines with Engineered Spectral Features (Algorithms A1s and A2s)

We also examined the performance of Algorithms A1r and A2r when the final classification steps were altered from the heuristic rule-based implementations to Support Vector Machines (SVMs). SVMs are a widely recognized approach to classification tasks [30]. An SVM is a supervised machine learning algorithm that works by identifying an optimal hyperplane in an augmented feature domain that separates observations into distinct classes. In this case, observations that fall on one or the other side of the hyperplane are classified as tremor present or absent. Importantly, the feature domain can be augmented with features derived via nonlinear functions (here, radial basis functions) in order to accommodate linearly non-separable classes in the original data. Here, we extracted the spectral features identified by each algorithm (summarized in Table 3) and used them as inputs to two separate SVMs with 5-fold cross-validation and radial basis function kernels.

#### 2.6.4. Modern Classifiers (Algorithms B1 and B2)

The final two algorithms (B1 and B2) were developed specifically for this study. They use basic preprocessing and spectral features together with well-established machine learning models to identify tremor in kinematic data.

##### Feature Extraction

In order to decouple tremulous movements from voluntary movements, the vector position of each kinematic marker on a given extremity is initially calculated as a measure of its instantaneous distance from the origin of the kinematic reference frame. This is carried out by calculating the Euclidean norm of the x, y, and z coordinates at all time instants, resulting in a single signal per sensor, as a function of time. The resulting signals are bandpass-filtered between 1 Hz and 20 Hz with a linear-phase finite impulse response (FIR) filter design using a hamming window of order 80. The signals are next decimated from 120 Hz to 40 Hz to further focus on the spectral range of interest. Next, the spectra of each sensor’s signal are estimated by using sliding windows of 3 s and 2.75 s overlapping with a 120-point discrete Fourier transform (DFT). The Welch power spectral density (PSD) estimation method with a Hamming window of 120 samples is used for PSD estimation, followed by a Gaussian-shaped moving average with a standard deviation of 1 Hz, to further smooth the spectra, sharpening the dominant frequencies and making them more distinguishable for the classifier. This results in 120 points of two-sided PSD with a spectral resolution of 0.33 Hz (40 Hz/120). The first 61 PSD values (corresponding to the DC component and one-sided spectrum) are used as the spectral feature vector of each sensor. The average feature vector calculated across all kinematic markers on a given extremity are then used as inputs to each of the classifiers described below.

##### B1: SVM Classification

In algorithm B1, the 61-point one-sided average spectral features were directly provided to an SVM as feature vectors. We considered SVM models with both linear and radial basis function (RBF) kernels. A standard stratified 5-fold cross validation scheme was performed by splitting the data into 5 non-overlapping splits, using 4 splits for training and the left-out split for validation. The stratification ensured that each fold retained approximately equal proportions of the two class labels.

##### B2: XGBoost Classification

In Algorithm B2, the 61-point one-sided average spectral features were directly provided to XGBoost as feature vectors. XGBoost is also a widely recognized approach to classification tasks [31]. XGBoost operates by iteratively constructing an ensemble of decision trees and refining them based on a specified loss function. The procedure for loading the features was analogous to the SVM process, again using stratified 5-fold cross-validation to ensure balanced representation across data splits. The classifier was configured to bypass label encoding, opting instead for the “logloss” evaluation metric. This probability-centric metric enables the future extension of the proposed scheme for estimating probabilities of tremulous events, instead of a binary decision.

### 2.7. Performance Metrics

We compared classifier performance based on the primary outcome F1 score, [12] as well as secondary outcomes Accuracy, Precision, Recall, and Specificity. F1 score is popular in binary classifiers because it considers both false positives and false negatives, providing a single performance measure [32]. It avoids misleading results from predicting all cases as one class. While sensitivity and specificity are crucial in clinical settings, the F1 score is widely used for comparing classifiers and was chosen as the primary outcome here.

We split the data into five separate 80/20 train/test folds, such that each fold contained nominally 1818 training and 454 test entries. With the exception of Algorithms A1r and A2, we then trained each of the algorithms on the training data in each fold and evaluated the performance on the test data within each fold. Because A1r and A2r did not require training, we simply report the average performance across folds for these two. We defined the outcome measures as follows:

(1) $$\begin{array}{cc} Accuracy & =\frac{TP+TN}{TP+TN+FP+FN} \end{array}$$

(2) $$\begin{array}{cc} Precision & =\frac{TP}{TP+FP} \end{array}$$

(3) $$\begin{array}{cc} Recall & =\frac{TP}{TP+FN} \end{array}$$

(4) $$\begin{array}{cc} Specificity & =\frac{TN}{TN+FN} \end{array}$$

(5) $$\begin{array}{cc} F1\;score & =2\times \frac{Precision\times Recall}{Precision+Recall} \end{array}$$

where TP (true positive) represents the total of successfully classified tremor-positive records, FP (false positive) represents the total number of misclassified tremor-negative records, TN (true negative) represents the total of successfully classified tremor-negative records, and FN (false negative) represents the total number of misclassified tremor-positive records.

### 2.8. Statistical Analyses

We calculated average F1 score and z-type 95% confidence intervals for each model, ranked them, and evaluated overlaps. Additionally, we created two linear mixed models to evaluate two specific hypotheses across all performance outcomes. First, we designed a linear mixed model to compare the performance of models A1s and A2s vs. A1r and A2r to assess whether modern classifier architectures resulted in improved performance vs. legacy architectures, while holding features constant. We implemented this model in “lmertest::lmer” in R software with a fixed effect for model type (legacy vs. modern), a fixed effect for outcome type (F1, Accuracy, Precision, Recall, and Specificity), and a random effect for fold. Next, we designed a linear mixed model to compare the performance of models B1 and B2 vs. all other models to assess whether these modern classifiers designed specifically for this study outperformed legacy architectures. This model also includes fixed effects for model type and outcome type and a random effect for fold.

### 2.9. SHAP Plots

Finally, we characterized the contributions of different frequency bands to classification with SHAP (SHapley Additive exPlanations) [33] plots derived from model B2. SHAP plots visually represent how much each feature contributes to the classification of each observation as one class or the other. This is analogous to the visual representation of factor loadings in familiar techniques like principal components analysis (PCA) but is adapted for nonlinear techniques like XGBoost.

## 3. Results

### 3.1. Characteristics of Annotations

The most frequent clinical annotation was “present”; however, clinicians used a range of qualitative labels to indicate tremor size. A description of the mapping between raw clinician-provided labels and dichotomized dataset labels is provided in Table 4. Clinician records for each patient comprised separate annotations of tremor presence in each of 16 separate extremities in each of multiple trials (mean ± SD, 11.1 ± 5.2; range, 1–25). Annotations most frequently referred to the hands (37%), although annotations for all extremities were present. The frequencies of appearance of various body extremities are described in Table 5. Chi-squared tests were used to identify significant differences in reporting frequencies between the arms and legs (*p* ≪ 0.001) and between the arms and head/torso (*p* ≪ 0.001).

### 3.2. Model Performance

**Figure 4:**
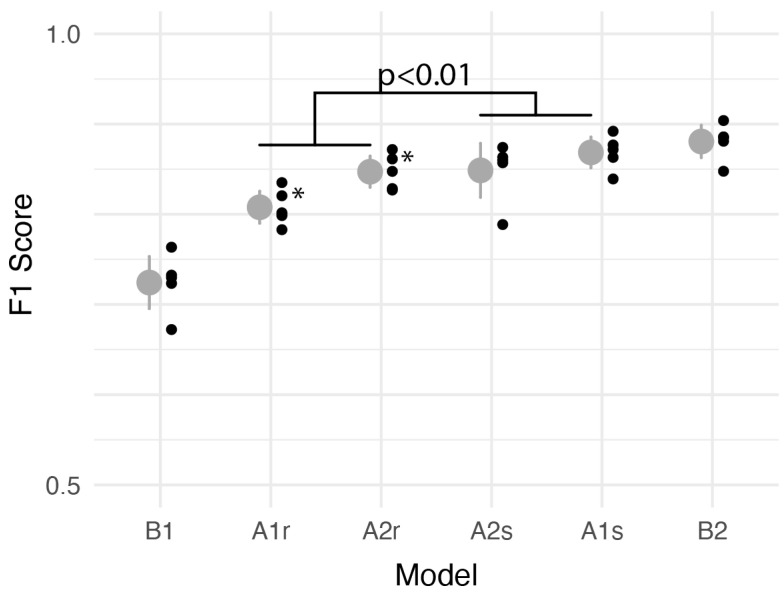
Comparison of *F1* score across models. Gray circles and lines represent average *F1* score ±95% CI across validation folds. Black circles represent performance on each fold. Models are ranked in ascending order of performance; models representing a statistically significant increase are designated with asterisks. A linear mixed model identified significantly improved performance associated with adding modern classifiers to legacy feature extraction algorithms (A1s and A2s vs. A1r and A2r). A linear mixed model comparing modern classifiers B1 and B2 to other classifiers identified no statistically significant effect.

**Figure 5:**
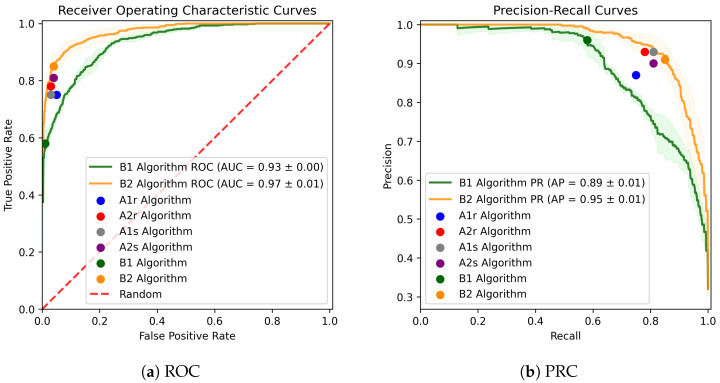
The average receiver operating characteristic (ROC) and precision-recall (PRC) curves for the SVM and XGBoost classifiers using spectral features of the spatial positions of the sensors. The shades correspond to ±1 standard deviations of each curve across the five-fold cross-validation. Colored dots illustrate average performance over five cross-validation folds.

All performance metrics for all models are reported in Table 6; F1 scores for each model are presented in rank order in Figure 4. The overall highest performance, as assessed with average F1 score across five cross-validation folds, was observed for the XGBoost classifier using generic spectral features, Algorithms B2. However, most classifiers performed well, and both legacy rule-based classifiers exhibited F1 scores >0.8. There was no statistically significant difference in performance between model B2 and the second-place model, A1s. Linear mixed models identified significant performance improvements when modern classifiers were added to legacy feature extraction algorithms (A1s and A2s vs. A1r and A2r; *p* < 0.01) but no difference between the classifiers created specifically for this study (B1 and B2) and the other classifiers (*p* = 0.57). Figure 5 compares the performance of Algorithms B1 and B2 with operating point information superimposed for the other algorithms. To ensure non-normal performance measures did not affect the results, we repeated the analysis using a Box–Cox transformation and found similar statistical significance patterns [34].

**Figure 6:**
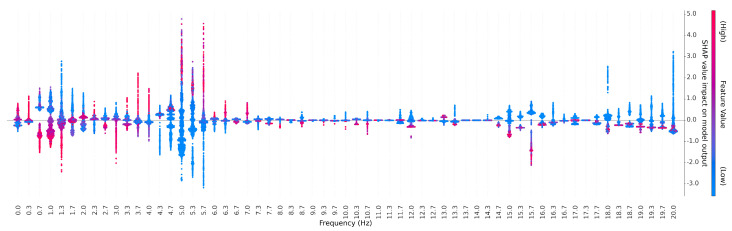
SHAP (SHapley Additive exPlanations) plot illustrating the contribution of each spectral feature across the Nyquist band to the tremor prediction results. Each column on the plot represents a specific feature’s contribution to the prediction. Positive SHAP values drive the model’s output towards the tremor class, while negative values drive towards the non-tremor class. The color intensity indicates the magnitude of the feature value, with red denoting high values and blue indicating low values. Notice the significance of the frequency range between 4.3 Hz and 7 Hz in identifying tremor. Frequencies below 3 Hz (corresponding to slow motions of the subject) are not informative for detecting tremor.

We further performed feature importance using SHAP values for all spectral features used as inputs to algorithms B1 and B2. The SHAP plots are shown in Figure 6. Unlike typical SHAP plots that are oriented vertically, in Figure 6, the feature importance is shown on the y axis and the features—characteristic frequencies within the kinematic data—are shown on the x axis, which allows for a visualization of the SHAP plots as a kind of spectrum. We note that a high activation of features in the frequency range between 4.3 Hz and 7.0 Hz were identified as significant for identifying tremor presence vs. absence (red), while a high activation of features in the frequency range between 0.7 Hz and 1.3 Hz wereidentified as significant in identifying tremor absence vs. presence (blue).

## 4. Discussion and Conclusions

The objective of this study was to assess the ability of several candidate processing pipelines to identify the presence or absence of tremor in kinematic data from movement disorders patients compared to expert ratings from movement disorders specialists. We found a high performance across multiple algorithms; the average F1 score was 0.83±0.06. Notably, the second-highest-performing algorithm (cross-validated F1=0.87) was Algorithm A1s, which was a version of the oldest algorithm in clinical use in our center that had been modified such that the manually engineered features were used as inputs to a modern SVM with radial basis function kernels to accommodate linearly non-separable data.

These results suggest some points that may be generally useful in settings with site-specific, legacy clinical decision support systems. In particular, in our clinic’s implementation, the existing algorithms A1r and A2r lacked a clear separation between feature identification and classification steps. We anticipate that this may be the case in other centers with site-specific, legacy systems. Refactoring legacy code to separate these two steps may provide an important opportunity to introduce updated classifier architectures into these systems without discarding the rich domain knowledge that is embedded in the derivation of engineered features. In our case, although some of the engineered features (e.g., dominant frequency) could be trivially discovered by classifiers with generic spectral features (like B1 and B2), other features (e.g., symmetry of the dominant spectral peak) reflect clinical domain expertise that automated searches could miss given limited training data. We anticipate this as a common issue and recommend that centers utilizing legacy data processing routines refactor their algorithms to distinguish between feature extraction and classification to address this potential limitation and enhance algorithm performance.

Further, when we visualized the Receiver-Operator Characteristic curves (Figure 5), we found that the clinical algorithms A1r and A2r were tuned to penalize false positive rate at the expense of some sensitivity in clinical use. Because these algorithms were originally without hyperparameter tuning, this was not performed intentionally on the part of the clinicians using these tools. Refactoring code could give clinicians the opportunity to tailor the balance of precision and recall to the clinical task at hand.

An analysis of SHAP plots revealed interesting information about the spectral composition of tremor. We noted that high activation of features in the frequency range between 4.3 Hz and 7.0 Hz was identified as significant for identifying tremor presence vs. absence (red), while high activation of features in the frequency range between 0.7 Hz and 1.3 Hz wasidentified as significant in identifying tremor absence vs. presence (blue) (Figure 6). This is consistent with the literature using various sensing modalities that have described tremor [4] as producing frequency band activity around 5 Hz, with voluntary movement producing lower frequency band activity below 3 Hz. In particular, these results show that a low frequency movement is not informative for detecting tremor; in fact, these frequencies have negative predictive value, suggesting that voluntary movements have the potential to be interpreted as false positives.

This was an informative result, as both clinical algorithms A1r and A2r were designed with features engineered to capture spectral information that was informative about tremor presence (presumably around 5 Hz) but imposed no penalties on low-frequency information that indicated that it was absent. This could be interpreted to mean that the original developers of these algorithms exhibited some cognitive confirmation or similar bias when designing the features to represent the aspects of the behavior they “knew about”, while neglecting equivalent kinematic information that was informative about the absence of tremor. The ability of modern ML to discover features may provide a unique opportunity to complement engineered features created with domain expertise.

### 4.1. Limitations

Our primary aim was to develop a generic tremor identification algorithm that could be used across extremities, behavioral tasks, and diagnoses in our center. Although the resulting algorithm is almost certainly not optimal in all settings, this approach generally aligns with clinical best practices and represents an important first step in the development of a comprehensive clinical decision support tool for tremor. However, this necessarily comes with the limitation that this tool may not be appropriate for all tremor identification tasks or patient populations. To investigate differential performance between diagnoses, we calculated diagnosis-specific F1 scores for PD, ET, and “other” using the highest-performing classifier, B1 (XGBoost). Performance for PD and ET was similar, with F1 scores (95% CI) of 0.89 (0.87–0.90) and 0.91 (0.88–0.94), respectively. Performance for other diagnoses was lower at 0.71 (0.67–0.76). These results suggest that while the classifier performs well for most clinic patients, caution is needed for rarer diagnoses. Some limitations due to the sample size are of note. First, a larger sample will be required to assess model performance in relatively infrequent conditions like dyskinesia or dystonia; these recordings comprise <2% of the current sample, preventing a reliable subgroup analysis. Similarly, a larger sample would provide more confidence in these models applied to extremities with less prevalent tremor. Finally, a larger sample would enable us to do comprehensive hyperparameter optimization, which was not available here, and to prevent patient data from being used across folds, which could reduce overfitting. A related but separate point is that our sample generally reflects the makeup of our database rather than the patient population as a whole. In particular, our clinic sees many more PD than ET patients due to the higher prevalence of functional neurosurgery in the PD cohort, even though ET is much more prevalent than PD. Further, our tremor assessment approach does not use scripted voluntary movements and weight application in order to isolate mechanical, volitional, and pathophysiological causes of tremor [35]; neither does it attempt, in this initial study, to quantify tremor size. For the above reasons, it will be important for other centers to consider carefully whether these results can be deployed in other centers without site-specific modifications.

### 4.2. Unique Contributions

Our method assesses tremor across the body, a unique capability. In a recent machine learning review on tremor applications, only 14% (5/37) explored body parts beyond the hands or distal arms [1]. This instrumentation is certainly convenient and almost certainly sufficient for tremor characterization with frequency [4] or amplitude [36,37]. However, we know that signal processing approaches like correlation across body regions provide additional diagnostic insight for discriminating, for example, parkinsonian from orthostatic tremors [35]. With full body data, end-to-end machine learning approaches (e.g., [12]) have significant potential to discover these and other features automatically. Other more subtle tremor features like distractibility [35] seem more likely to be characterized in full body data. Further, our testing approach imposes few, if any, constraints on the participants’ natural movements. This complicates data analysis compared to methods that confine movements to a single plane, (e.g., [16]) but may improve external validity.

### 4.3. Conclusions

Here, we sought to assess the ability of several candidate processing pipelines to identify the presence or absence of tremor in kinematic data from movement disorders patients compared to expert ratings from movement disorders specialists. We found that many solutions offered acceptable performance. The best individual-performing algorithm was a modernization of one of the oldest algorithms in constant clinical use in our center. In general, updating legacy clinical decision support systems to incorporate modern machine learning classifiers may result in better-performing tools and associated decreases in provider time and improved outcomes.

## Figures and Tables

**Table 1:** Clinical and demographic characteristics of the study sample. The dataset also contains records of two additional patients (*B* and *Q*) for whom clinical and demographic information was unavailable.

Variable	Value
N	50
Sex	
Male	32 (64%)
Female	18 (36%)
Age, years	
Mean (SD)	66 (12)
Range	36–83
Race	
White	46 (92%)
Black	2 (4%)
Unknown/Not Reported	2 (4%)
Ethnicity	
Non-Hispanic or Latino	41 (82%)
Hispanic or Latino	2 (4%)
Unknown/Not Reported	7 (14%)
Primary Diagnosis	
Parkinson’s disease (PD)	29 (56%)
Essential tremor (ET)	14 (27%)
Dystonia	5 (10%)
Enhanced physiological tremor	1 (2%)
Functional tremor	1 (2%)
PD duration, years	
Mean (SD)	9.9 (5.0)
Range	1–23
MDS-UPDRS-III [6]	
Mean (SD)	38.2 (16.7)
Range	5–76
ET duration, years	
Mean (SD)	25.4 (14.1)
Range	5–53
TETRAS (ADL) [25]	
Mean (SD)	5.5 (11.6)
Range	0–37
TETRAS (P) [25]	
Mean (SD)	27.2 (7.3)
Range	16–39.5

**Table 2:** Comparison of tremor identification algorithms. All algorithms operate on spectral features of kinematic data.

Algorithm	Kinematic Data	Features	Aggregation	Classifier
A1r	Velocity	Engineered	Winner-take-all	Rule-Based
A1s				SVM
A2r	Position	Engineered	Average	Rule-Based
A2s				SVM
B1	Position	Generic Spectral	Average	SVM
B2	Position	Generic Spectral	Average	XGBoost

**Table 3:** Spectral features calculated by clinical algorithms A1r/s and A2r/s.

Algorithm	Feature	Description
A1r/s	F_CENTER	Tremor frequency (Hz)
	AMPLITUDE_MM_P_S	Tremor amplitude (mm/s)
	BW	3 dB bandwidth (Hz)
	HI_F	Left frequency border (Hz)
	LO_F	Right frequency border (Hz)
	MAX_POWER	Maximum power level of the power spectrum (dB/Hz)
	HI_POWER	Power level at right frequency border (dB/Hz)
	LO_POWER	Power level at left frequency border (dB/Hz)
	RELATIVE_POWER	Proportion of total power
A2r/s	F_CENTER	Tremor frequency (Hz)
	AMPLITUDE_MM	Tremor amplitude (mm)
	PROMINENCE	Peak prominence (mm)
	WIDTH	Peak width (Hz)

**Table 4:** Mapping between clinician-provided labels and training labels in training data. The “Other” label aggregates annotations with fewer than 10 observations and annotations for which no indicator size was provided (e.g., “RH tremor”).

Raw Label	Binary Label in Dataset	
	Absent	Present
Absent	1476	0
Dystonia, dyskinesia, or other abnormal posture or movement	68	0
Present	0	288
Not much	0	24
Very slight or very trace	0	10
Slight or trace	0	78
Intermittent	0	10
Mild	0	121
Mild to moderate	0	34
Moderate	0	55
Moderate to severe	0	17
Significant	0	32
Severe	0	17
Other, or no indicator of size	0	42

**Table 5:** Frequency table of tremor annotations.

Extremity	Absent	Present	Total
Head/Torso	296	56	352
Head	76	52	
Shoulders	71	2	
Thorax	78	1	
Pelvis	71	1	
Arms	767	569	1336
L_Dist_Arm	73	5	
R_Dist_Arm	72	1	
L_Hand	237	280	
R_Hand	242	279	
L_Prox_Arm	71	3	
R_Prox_Arm	72	1	
Legs	481	103	584
L_Dist_Leg	81	21	
R_Dist_Leg	81	8	
L_Foot	76	30	
R_Foot	81	15	
L_Prox_Leg	81	21	
R_Prox_Leg	81	8	

**Table 6:** Comparison of mean algorithm performance for primary and secondary outcomes. Performance is reported as Mean (SD) across five separate data folds. The highest performance for each performance outcome is indicated in bold. A1r, A2r, A1s, and A2s are reference algorithms and do not have degrees of freedom to form ROC and PRC curves.

Metric	A1r	A2r	A1s	A2s	B1	B2
F1	0.81 (0.02)	0.85 (0.02)	0.87 (0.02)	0.85 (0.03)	0.72 (0.03)	**0.88 (0.02)**
Accuracy	0.89 (0.01)	0.91 (0.01)	0.92 (0.01)	0.91 (0.01)	0.86 (0.02)	**0.93 (0.02)**
Precision	0.87 (0.01)	0.93 (0.01)	0.93 (0.01)	0.90 (0.03)	**0.96 (0.02)**	0.91 (0.03)
Recall	0.75 (0.03)	0.78 (0.02)	0.81 (0.03)	0.81 (0.04)	0.58 (0.04)	**0.85 (0.02)**
Specificity	0.95 (0.01)	0.97 (0.01)	0.97 (0.01)	0.96 (0.01)	**0.99 (0.01)**	0.96 (0.02)
AUROC	–	–	–	–	0.93 ± 0.00	**0.97 ± 0.01**
AUPRC	–	–	–	–	0.89 ± 0.01	**0.95 ± 0.01**

**Figure A1:**
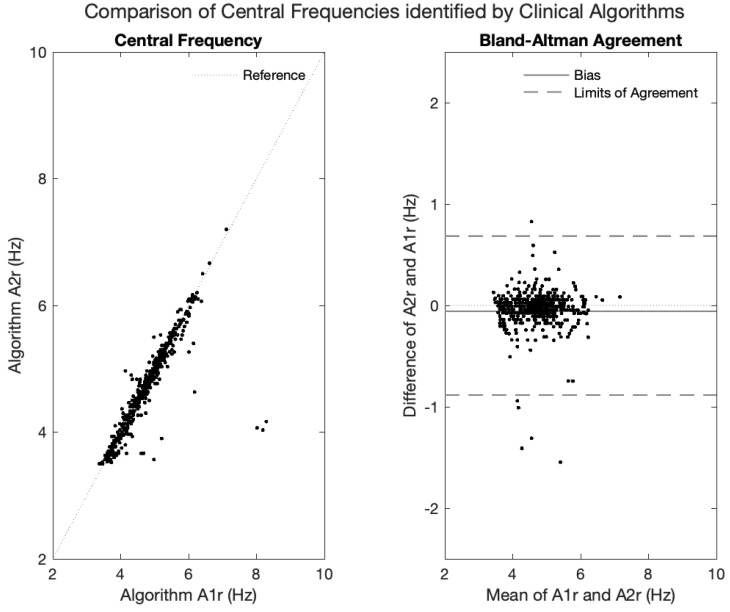
Comparison of tremor frequencies identified by clinical algorithms A1r and A2r.

**Figure A2:**
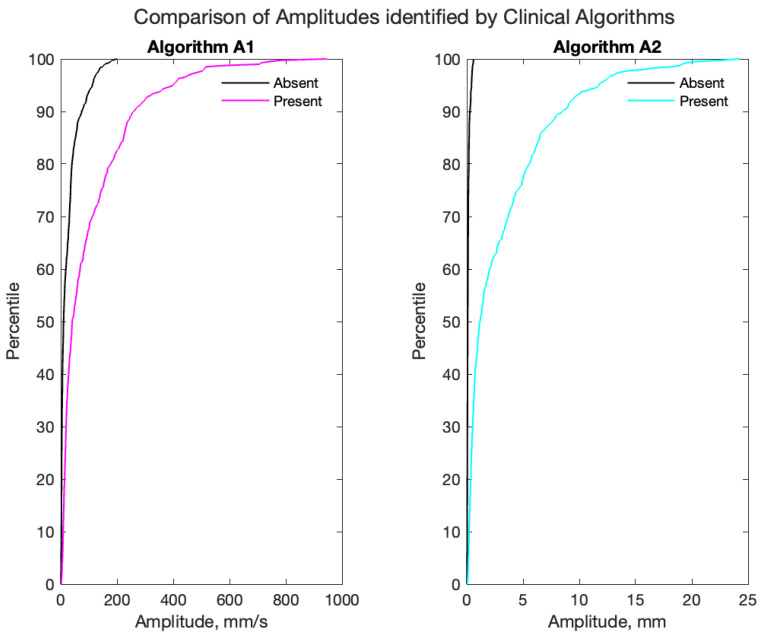
Comparison of tremor amplitudes identified by clinical algorithms A1r and A2r, stratified by ground-truth labels of tremor presence or absence.

## Data Availability

Data are available upon reasonable request from the corresponding author.

## Abbreviations

The following abbreviations are used in this manuscript:

CPT	Current Procedural Terminology
ET	Essential tremor
FTM	Fahn–Tolosa–Marin Tremor Rating Scale
MDS-UPDRS-III	Movement Disorder Society-Unified Parkinson’s Disease Rating Scale Part III
PD	Parkinson’s disease
SHAP	SHapley Additive exPlanations
SVM	Support vector machine
TETRAS	The Essential Tremor Rating Scale

## Appendix A. Comparison of Tremor Features Identified by Algorithms A1r and A2r

We performed some additional analyses to compare the tremor features identified by clinical algorithms A1r and A2r. We compared tremor frequencies identified by clinical algorithms A1r and A2r using a Bland–Altman approach [38]. Because each of the clinical algorithms produced an estimate of tremor amplitude whether or not a tremor was detected, we compared the ranges of amplitudes obtained when tremor was present or absent according to ground-truth labels with two-sample Kolmogorov–Smirnov tests.

Overall, Algorithms A1r and A2r identified very similar central tremor frequency estimates, with average values of 4.8±1.0 Hz and 4.7±0.6 Hz, respectively. The Bland–Altman analysis between the results of the two algorithms identified a bias of −0.1 Hz between the two algorithms, with 95% limits of agreement (−9.0, 0.7) Hz (Figure A1).

With both algorithms, the range of identified amplitudes for which tremor was rated present according to expert labels had some overlap with the range of amplitudes for which tremor was rated absent. This suggests that a simple amplitude-based threshold would be insufficient to discriminate tremor presence using either approach. With Algorithm A1r, the average amplitude when tremor was present was [102.2±13.40, 1.9–944.7] mm/s ([Mean ± SD, range]) compared to [26.4±35.1, 0.4–199.0] mm/s when tremor was absent. With Algorithm A2r, the average amplitude when tremor was present was [3.07±3.00, 0.3–24.3] mm compared to [0.13±0.12, 0.01–0.59] mm when tremor was absent. The cumulative densities identified by both algorithms showed separation between cases labeled as tremor absent and present and highly significant (*p*≪ 0.001) two-sample Kolmogorov–Smirnov tests. A visual inspection of cumulative amplitude distributions (Figure A2) for the two algorithms suggests that A2r provided better separation, although this could not be compared directly due to the different units used by the algorithms.

## Appendix B. Additional Dataset Details

This section provides some additional details of the data format and coding scheme. During each individual behavioral test, the laboratory 3D motion capture system records the instantaneous position of all kinematic markers on the body (typically 60) and exports these data to a standard **.trc* tabular data format with some minimal header information. A typical *.trc* file for a 30 second recording at 120 Hz comprises 3600 rows (30 s × 120 Hz) and 180 columns (60 markers × 3 axes) of kinematic data. Because each file includes data from markers on different extremities, for which tremor may be absent or present on a given trial, the columns of the **.trc* file corresponding to markers on each extremity must be separated prior to analysis. Our clinical data processing pipeline maintains the mappings between kinematic markers and extremities in an **.xml* file (markers.xml). To avoid the burden of parsing these files, the data supplied with the paper are provided in two different formats. Each de-identified *.trc* file is provided as originally exported, as well as divided into separate *.csv* files for each body extremity in the accompanying dataset. These files are compatible with standard Python, R, Matlab, or similar software libraries. Summaries of the contents of example files are provided in Table A1. Descriptions of kinematic marker locations are provided in Table A3, Table A4 and Table A5.

Information about the testing condition used during each recording is provided as part of the individual file names, using the nomenclature provided in Table A2. For example, the file data/HH/std-arms-extended1-TP.trc designates participant HH standing with arms extended forward along the X axis of the laboratory Figure 1. In pointing and spiral movement tasks, the suffix right or left, or 1 or 2, is appended to the filename denoting the extremity involved. In some cases, extra motor or cognitive tasks were introduced to intensify tremor provocation, with supplementary information appended to the base task codes.

**Table A1:** Example file descriptions and load methods. In some cases .trc files contain additional columns with derived variables that should be ignored.

Example File	*std-arms-extended1-TP.trc*	*std-arms-extended1-TP/R_Hand.csv*
**Description**	Tabular data with header exported by motion capture software	Portion of .trc file corresponding to extremity R_Hand
**Columns**		
Number	182	Variable
Contents	Index	Index
	Time (seconds)	Time (seconds)
	Whole-body kinematic marker data arranged as x, y, z	Extremity kinematic marker data from R_Hand arranged as x, y, z
**Load methods**	loadTrc.m	readtable.m
		pandas.read_csv
		readr::read_csv

**Table A2:** Nomenclature for behavioral tasks employed in testing.

Code	Task
sit-rest	Seated with arms at sides
sit-arms-extended	Seated, with arms extended anteriorly and parallel to the floor
sit-UEopp	Seated, with arms in a “T” pose parallel to the ground with fingers of each hand opposed
sit-point	Seated, performing a finger-to-nose pointing task with the indicated extremity (*right/1* or *left/2*)
sit-spiral	Seated, performing a spiral movement with the indicated extremity (*right/1* or *left/2*)
std-rest	Standing with arms at sides
std-arms-extended	Standing, with arms extended out parallel to the ground
std-UEopp	Standing, with arms in a “T” pose parallel to the ground with fingers of each hand opposed
walk-thru	Comfortable walking from one end to the other of the motion capture space
TUG	Sequential “timed up and go” walking tasks

**Table A3:** Kinematic marker descriptions for markers on the trunk. Markers that appear on both sides of the body are listed for the right side only and are coded beginning with “R”. Replacing this character with “L” will designate the corresponding marker on the left side of the body.

Extremity	Marker Code	Description
Head	Front_Head	Center of forehead, on cap
	JAW	Mental protuberance
	Jaw	Mental protuberance
	RBHD	Right back head, on cap
	RFHD	Right front head, on cap
	Rear_Head	Rear of head, on cap
	TopHead	Top of head
	Top_Head	Top of head
Shoulders	C7	Seventh cervical vertebra
	RBAK	Right scapula (asymmetry marker)
	RSHO	Right acromioclavicular joint
	R_Shoulder	Right acromion process
	STRN	Xiphoid process
Pelvis	LASI	Left anterior superior iliac spine
	RASI	Right anterior superior iliac spine
	RIC	Right iliac crest
	RPSI	Right posterior superior iliac spine
	R_ASIS	Right anterior superior iliac spine
	V_Sacral	Sacrum
Thorax	CLAV	Clavicular notch
	R_Clavicle	Right clavicle
	R_Scap_Inf	Right scapula inferior angle
	R_Scapula	Right supraspinous fossa
	T10	10th thoracic vertebra

**Table A4:** Kinematic marker descriptions for markers on the arms. Markers that appear on both sides of the body are listed for the right side only and are coded beginning with “R”. Replacing this character with “L” will designate the corresponding marker on the left side of the body.

Extremity	Marker Code	Description
R_Dist_Arm	RFRM	Lateral surface of forearm
	RWRA	Radial side of wrist
	RWRB	Ulnar side of wrist
	R_Forearm	Lateral surface of forearm
	R_Radius	Right styloid process of radius
	R_Ulna	Mid region of ulna
R_Hand	RFIN	Third finger, first metacarpal joint
	RFINGM2	Third finger, second metacarpal joint
	RFINGM3	Third finger, most distal segment
	RTHM1	Thumb, first metacarpal
	RTHM2	Thumb, second metacarpal
	RTHM3	Thumb, most distal segment
	R_Finger3_M1	Third finger, first metacarpal joint
	R_Finger3_M2	Third finger, second metacarpal joint
	R_Finger3_M3	Third finger, most distal segment
	R_Hand	Radial surface of wrist
	R_Thumb_M1	Thumb, first metacarpal
	R_Thumb_M2	Thumb, second metacarpal
	R_Thumb_M3	Thumb, most distal segment
	R_Wrist	Radial surface of wrist
R_Prox_Arm	RELB	Right lateral epicondyle
	R_BicepsLateral	Lateral surface of upper arm
	R_Biceps_Lateral	Lateral surface of upper arm
	R_Elbow	Right lateral epicondyle
	R_Elbow_Medial	Right medial epicondyle

**Table A5:** Kinematic marker descriptions for markers on the legs. Markers that appear on both sides of the body are listed for the right side only and are coded beginning with “R”. Replacing this character with “L” will designate the corresponding marker on the left side of the body.

Extremity	Marker Code	Description
R_Dist_Leg	RANK	Lateral aspect of ankle
	RANKM	Medial aspect of ankle
	RTIB	Midpoint of tibia
	R_Ankle	Lateral aspect of ankle
	R_Ankle_Medial	Medial aspect of ankle
	R_Shank	Midpoint of tibia
R_Foot	RFTM	Dorsal/medial surface of foot midway between ankle and toe
	RHEE	Distal surface of heel
	RTOE	Third metatarsal
	R_Hallux	Dorsal surface of big toe
	R_Heel	Distal surface of heel
	R_MedFoot	Dorsal/medial surface of foot midway between ankle and toe
	R_Toe	Third metatarsal
R_Prox_Leg	RKNE	Lateral aspect of flexion–extension axis of knee
	RKNEM	Medial aspect of flexion–extension axis of knee
	RTHI	Upper lateral 1/3 surface of thigh
	R_Knee	Lateral aspect of flexion–extension axis of knee
	R_Knee_Medial	Medial aspect of flexion–extension axis of knee
